# Molecular Engineering of Fungal GH5 and GH26 Beta-(1,4)-Mannanases toward Improvement of Enzyme Activity

**DOI:** 10.1371/journal.pone.0079800

**Published:** 2013-11-22

**Authors:** Marie Couturier, Julia Féliu, Sophie Bozonnet, Alain Roussel, Jean-Guy Berrin

**Affiliations:** 1 INRA, UMR1163, Laboratoire de Biotechnologie des Champignons Filamenteux, Polytech Marseille, Marseille, France; 2 Aix Marseille Université, Polytech Marseille, Marseille, France; 3 Université de Toulouse; INSA, UPS, INP; LISBP, Toulouse, France; 4 INRA, UMR792, Ingénierie des Systèmes Biologiques et des Procédés, Toulouse, France; 5 CNRS, UMR5504, Toulouse, France; 6 Architecture et Fonction des Macromolécules Biologiques, UMR7257, CNRS, Aix Marseille Université, Marseille, France; University of South Florida College of Medicine, United States of America

## Abstract

Microbial mannanases are biotechnologically important enzymes since they target the hydrolysis of hemicellulosic polysaccharides of softwood biomass into simple molecules like manno-oligosaccharides and mannose. In this study, we have implemented a strategy of molecular engineering in the yeast *Yarrowia lipolytica* to improve the specific activity of two fungal endo-mannanases, *Pa*Man5A and *Pa*Man26A, which belong to the glycoside hydrolase (GH) families GH5 and GH26, respectively. Following random mutagenesis and two steps of high-throughput enzymatic screening, we identified several *Pa*Man5A and *Pa*Man26A mutants that displayed improved kinetic constants for the hydrolysis of galactomannan. Examination of the three-dimensional structures of *Pa*Man5A and *Pa*Man26A revealed which of the mutated residues are potentially important for enzyme function. Among them, the *Pa*Man5A-G311S single mutant, which displayed an impressive 8.2-fold increase in *k_cat_*/K_M_ due to a significant decrease of K_M_, is located within the core of the enzyme. The *Pa*Man5A-K139R/Y223H double mutant revealed modification of hydrolysis products probably in relation to an amino-acid substitution located nearby one of the positive subsites. The *Pa*Man26A-P140L/D416G double mutant yielded a 30% increase in *k_cat_*/K_M_ compared to the parental enzyme. It displayed a mutation in the linker region (P140L) that may confer more flexibility to the linker and another mutation (D416G) located at the entrance of the catalytic cleft that may promote the entrance of the substrate into the active site. Taken together, these results show that the directed evolution strategy implemented in this study was very pertinent since a straightforward round of random mutagenesis yielded significantly improved variants, in terms of catalytic efiiciency (k_cat_/K_M_).

## Introduction

Hemicellulose is a combined designation of a diverse set of abundant non-crystalline carbohydrate polymers, among which mannans are the major component in softwoods [1] and are also present in certain plant seeds [2]. Mannans comprise molecules constituted either by a backbone of β-1,4-linked D-mannose residues, known as mannan, or by a heterogeneous combination of β-1,4-D-mannose and β-1,4-D-glucose units, termed glucomannan. Both can be decorated with α-1,6-linked galactose side chains, and these polysaccharides are referred to as galactomannan and galactoglucomannan, respectively. According to Salmén [3] softwood glucomannans are incorporated into aggregates of cellulose, i.e., they are arranged in parallel with cellulose fibrils, to which they are tightly connected.

Mannans are hydrolyzed by the coordinated action of several types of glycoside hydrolases (GH) among which endo-β-1,4 mannanases (EC 3.2.1.78) are the key enzymes that depolymerize the mannan backbone. They are encountered in families GH5, GH26 and GH113 in the CAZy database [4], [5]. Beta-mannanases are useful in several industrial processes such as reduction of viscosity of coffee extracts [6] or biobleaching of softwood Kraft pulp [7] and it is now acknowledged that they will become increasingly important for the biorefining of lignocellulose, especially from softwood biomass [8]–[11]. In the biorefinery process, enzymatic hydrolysis of lignocellulosic biomass is one of the major bottlenecks due to the recalcitrance of the plant cell wall and the high cost of enzymes, mainly due to the fact that large amounts are required to breakdown lignocellulose to fermentable sugars [12]–[14].

Despite the fact that mannanases are largely exploited in biotechnological applications, only a few studies have so far reported on the improvement of mannanase properties using molecular engineering [15], [16]. Directed evolution is an important tool for improving critical traits of biocatalysts for industrial applications [17]. Recent advances in mutant library creation and high-throughput screening have greatly facilitated the engineering of biocatalysts but to date only few studies describe improvement of biomass-degrading enzymes using molecular evolution [18]–[20]. The major problem with most directed evolution experiments on biomass-degrading enzymes is the setup of high-throughput assays [17], [21], [22] since it is problematic to measure enzyme activities towards insoluble (hemi) cellulosic substrates.

In this study, a random mutagenesis strategy was used to generate variants of two endo-mannanases from the ascomycete fungus *Podospora anserina* that belong to the glycoside hydrolase (GH) families GH5 and GH26 (*Pa*Man5A and *Pa*Man26A, respectively) in order to improve their activity towards galactomannan. To evaluate the activity of mannanase variants produced in the *Yarrowia lipolytica* expression host, an in-house high-throughput method based on the reducing sugar assay [23] was adapted to assay mannanase activity in liquid culture. The results are interpreted in the lights of the three-dimensional structures of both fungal mannanases [24].

## Results and Discussion

### *Pa*Man5A and *Pa*Man26A heterologous expression in *Yarrowia lipolytica*

**Figure 1:**
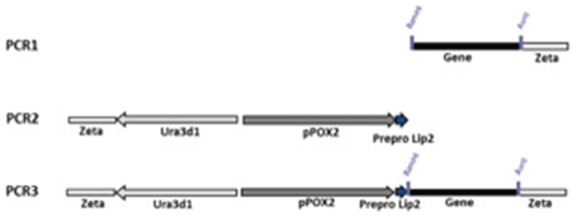
Error-prone PCR strategy used in the study. PCR1: error prone-PCR performed on *paman5a* (HM357135) and *paman26a* (HM357136); PCR2: PCR without mutation performed on Ura3d1 (selection marker), pPOX2 (inducible promotor of acyl-coA oxidase 2) and prepro Lip2 (secretion signal sequence); PCR3: overlapping PCR to reconstruct the entire sequence between zeta platforms. Primers used are listed in Table 3.

The yeast *Y. lipolytica* was chosen as host to perform molecular engineering of *Pa*Man5A and *Pa*Man26A since a very high reproducibility in protein expression level was demonstrated [25]. The two genes encoding *Pa*Man5A and *Pa*Man26A were inserted into the *Y. lipolytica* expression vector in frame with the yeast preprolip2 secretion peptide under the control of the oleic acid-inducible promoter *POX2* (Figure 1). Positive transformants selected on plates containing galactomannan were able to produce functional *Pa*Man5A and *Pa*Man26A enzymes to a level of 10.4±0.2 and 11.2±0.6 U.ml^−1^ in shake flasks, respectively. The culture conditions enabling the secretion of wild-type *Pa*Man5A and *Pa*Man26A were miniaturized in 96-well plates as described in [26] to facilitate the set-up of the high-throughput screening procedure. The mean activities calculated from repeated experiments were 1.78±0.14 and 1.24±0.15 U.ml^−1^ of culture for *Pa*Man5A and *Pa*Man26A, respectively (coefficient of variation [CV] of 7.7 and 12.2%, respectively), which is adequate for excluding false positives variants. The secretion yields of *Pa*Man5A and *Pa*Man26A in deep-well microplates were 6- and 9-fold lower than in shake flask cultures, which is probably due to a better oxygenation of cultures in flasks than in deep-well plates where the ratio volume of culture/volume of container was of 1/2 instead of 1/10. However, levels of mannanase production were in both cases sufficient to deploy a high-throughput screening campaign.

### Construction of first-round mutagenesis libraries and screening

**Figure 2:**
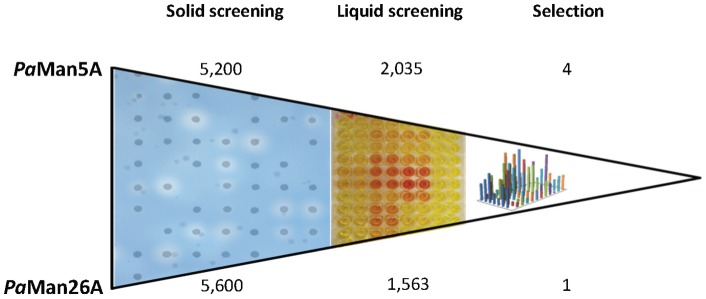
Screening strategy and mutant selection. The number of variants screened at each step is indicated at the top (*Pa*Man5A) and at the bottom (*Pa*Man26A) of the diagram.

The objective of the directed evolution approach was to improve enzymatic activities of *Pa*Man5A and *Pa*Man26A, two endo-mannanases from *Podospora anserina*. The error-prone PCR mutagenesis round was carried out using the wild type genes as templates (GenBank HM357135 and HM357136 for *paman5a* and *paman26a*, respectively) and with the conditions appropriate to reach a number of mutations in a range of 2 to 5 mutations per kb. Indeed, a very low number of mutations per kb (i.e., below one mutation per kb) would result in too many active variants with unchanged activity, and a high number of mutations per kb would result in too many inactive clones as reported in [17] (>70% inactive clones for a mutation rate of 10 mutation per kb). In our study, subcloning and sequence analysis of a representative set of mutated genes from each library revealed that the mutation rates were in the desired range (i.e. 2.6 and 4.5 mutations per kb for *Pa*Man5A and *Pa*Man26A, respectively). Figure 2 shows a summary of the libraries screening and the number of variants selected at each step. *Y. lipolytica* transformation yielded about 5,200 and 5,600 clones for *Pa*Man5A and *Pa*Man26A, respectively. As reported in other studies [20], [27], [28], 5,000 variants was considered as a reasonable size of library to identify mutants displaying improved characteristics. The first step of the screening consisted in the selection of active clones on solid medium containing AZO-dyed galactomannan. Since we were looking for an activity improvement that was expected to be rather modest, halo-producing clones could not be screened only by comparing the size of halos but required a second step of screening. Culture-based screening was performed using our in-house robotic platform with about 2,000 and 1,500 mutants for *Pa*Man5A and *Pa*Man26A, respectively (Figure 2). Since the screening capability of this high-throughput system is 15 plates per day, ∼1,400 variants could be analysed in one day and the defined screening job (3,500 variants) was finished within 3 days. Among the best-performing clones that were further tested to confirm the enhancement of activity in liquid cultures, we finally selected four mutants of *Pa*Man5A and one mutant of *Pa*Man26A that displayed improved activity beyond the CV of wild-type mannanases (7.7 and 12.2%, respectively). The *Y. lipolytica* strain expressing the best *Pa*Man26A variant displayed an increase of activity towards galactomannan of 147% compared to the strain expressing wild-type *Pa*Man26A that corresponded to 12 CV. The *Y. lipolytica* strains expressing selected *Pa*Man5A variants displayed also increased activity between 8 and 46% compared to the strain expressing wild-type *Pa*Man5A that corresponded to 1.1 to 6 CV. Each of the mannanase-mutant genes was amplified from genomic DNA, subcloned and sequenced. As a result, one single (*Pa*Man5A-G311S), two double (*Pa*Man5A-K139R/Y223H, *Pa*Man26A-P140L/D416G) and two triple mutants (*Pa*Man5A-V256L/G276V/Q316H, *Pa*Man5A-W36R/I195T/V256A) were created. Results are summarized in Table 1.

**Table 1:** Mannanase activity of selected *Y. lipolytica* variants.

Enzyme	Activity (U.ml^−1^)	CV (%)	Activity Improvement (%)
*Pa*Man26A wt	1.24±0.15	12.2	-
*Pa*Man26A-P140L/D416G	-	-	147
*Pa*Man5A wt	1.78±0.14	7.7	-
*Pa*Man5A-V256L/G276V/Q316H	-	-	46
*Pa*Man5A-W36R/I195T/V256A	-	-	9
*Pa*Man5A-K139R/Y223H	-	-	20
*Pa*Man5A-G311S	-	-	11

The mannanase activity was measured at 40°C in sodium acetate buffer 50 mM, pH 5.2 using 1% (w/v) galactomannan. The coefficient of variation (CV) was defined as the ratio of the standard deviation to the mean and was calculated for each of the wild-type enzymes. wt, wild type.

### Mutant production in *P. pastoris* and biochemical characterization

For mutant enzymes production, we used the *P. pastoris* expression system because (i) it yields higher expression levels than *Y. lipolytica* [29] and (ii) wild-type *Pa*Man5A and *Pa*Man26A were already successfully expressed to high yields in *P. pastoris* [10]. The ratio of mannanase production yields obtained in the culture medium with *Y. lipolytica* and *P. pastoris* was roughly 1∶10. All of the mutant enzymes were successfully produced in *P. pastoris* with yields of approximately 1 g per liter of culture and purified taking advantage of the (His)_6_-tag. SDS-PAGE analysis of the purified mutants compared to the wild type enzymes revealed similar apparent molecular masses (data not shown). Isoelectrofocusing analysis revealed that all of the mutants displayed similar pIs compared to wild-type enzymes except for the *Pa*Man5A-W36R/I195T/V256A mutant that exhibited an increase of 0.3 unit (Figure S1). This increase is likely to be due to the incorporation of an arginine residue. Circular dichroism analysis of all selected mutants was also carried out to check the relative content of secondary structures in each mutant. *Pa*Man26A and *Pa*Man5A variants displayed the same profile compared to parental enzymes, suggesting that the folding of mutants were similar to the parental enzymes (data not shown). Regarding the pH and temperature profiles, no significant difference was observed between variants and parental enzymes (data not shown).

### Kinetic parameters of mutants toward galactomannan

Each mutant was characterized using High Performance Anion Exchange Chromatography – Pulsed Amperometric Detection (HPAEC-PAD) to assess its ability to hydrolyze galactomannan and manno-oligosaccharides: mannopentaose (M_5_) and mannohexaose (M_6_) (Table 2). Regarding the M_5_ and M_6_ hydrolysis, *Pa*Man26A-P140L/D416G exhibited 100% and 30% increase of *k*_cat_/K_M_ toward M_5_ and M_6_, respectively. *Pa*Man5A-V256L/G276V/Q316H and *Pa*Man5A-W36R/I195T/V256A exhibited same hydrolysis profile as *Pa*Man5A at 10 min or 20 min manno-oligosaccharide hydrolysis (data not shown) and therefore *k*_cat_/K_M_ were considered unchanged and not determined. *Pa*Man5A-K139R/Y223H revealed a decrease of *k*_cat_/K_M_ of about 35% and 11% respectively. *Pa*Man5A-G311S displayed an increased *k*_cat_/K_M_ of about 37% and 12% towards M_5_ and M_6_, respectively.

**Table 2:** Kinetic constants of wild-type enzymes and selected variants toward galactomannan, mannohexaose (M_6_) and mannopentaose (M_5_).

	Galactomannan			M_6_	M_5_	
Enzyme	K_M_ (mg.ml^−1^)	*k_cat_* (min^−1^)	*k*_cat_/K_M_ (mg^−1^.ml.min^−1^)	*k*_cat_/K_M_ (mg^−1^.ml.min^−1^)		
*Pa*Man26A wt	2.4±0.3	3356±159	1413±155	7.6×10^5^		3.8×10^5^
26-P140L/D416G*	2.1±0.2	3860±80	1849±148	1.0×10^6^		7.8×10^5^
*Pa*Man5A wt	11.5±1.5	1674±138	147±19	3.0×10^6^		9.2×10^5^
5-V256L/G276V/Q316H	7.8±1.6	1493±167	197±38	ND		ND
5-W36R/I195T/V256A*	16.2±2.5	4199±438	263±41	ND		ND
5-K139R/Y223H*	6.7±0.5	1655±60	248±17	2.7×10^6^		6.8×10^5^
5-G311S*	1.5±0.4	1781±134	1247±292	3.4×10^6^		1.2×10^6^

The kinetic parameters were determined at 40°C in sodium acetate buffer 50 mM, pH 5.2 as described in the Methods section. Paired t test was used to compare the kinetic parameters of mutants versus native enzyme. The difference was considered statistically significant when *p*<0.05 (*). wt, wild type.ND: not determined.

Regarding the K_M_ apparent values, all of the mutants displayed improved apparent affinity for galactomannan. Although turn-over numbers of almost all of the mutant enzymes were similar to wild-type enzymes, the *Pa*Man5A-W36R/I195T/V256A mutant displayed a 2.5-fold increase of turn-over toward galactomannan resulting in an overall catalytic efficiency improved by 1.8-fold. In terms of catalytic efficiency, the best-performing mutant was the *Pa*Man5A-G311S mutant for which the single amino-acid substitution led to a 8.2-fold increase in *k*_cat_/K_M_ due to a drastic improvement of K_M_ whereas the *k*_cat_ remained unchanged. Other *Pa*Man5A mutants exhibited also to a lower extent increased *k*_cat_/K_M_ towards galactomannan, i.e., between 32 and 79% improvement. The *Pa*Man26A-P140L/D416G mutant, which is the unique mutant selected from the 5,600 *Pa*Man26A variants screened, displayed an improved *k*_cat_/K_M_ of approximately 30%. All of the mutants were subjected to a paired t-test in comparison with the corresponding native enzyme. Three out of four *Pa*Man5A mutants and the *Pa*Man26A mutant revealed *p*-values below 0.05 for galactomannan hydrolysis and were therefore considered statistically different from their native counterpart (Table 2).

Only sparse studies have previously led to the identification of mutants displaying such increase in catalytic efficiency. For example, several endoglucanase mutants with *k*_cat_/K_M_ improvement between 15 and 80% were generated after three rounds of mutagenesis [26]. Song *et al* [20] identified several xylanase mutants with *k*_cat_/K_M_ towards xylan increased in a range of 20 to 50% after several rounds of evolution.

### Structure-function relationships of identified variants

Examination of the recently solved three-dimensional structures of *Pa*Man5A and *Pa*Man26A [24] revealed that some of the mutated residues are potentially important for enzyme function.

**Figure 3:**
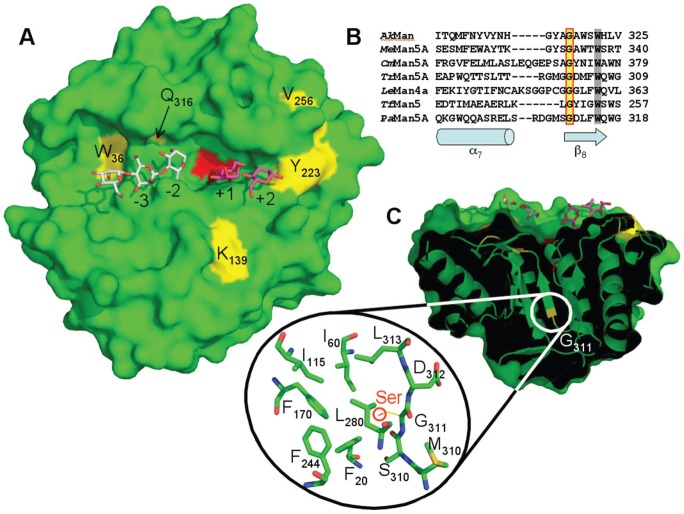
Structural view of *Pa*Man5A (PDB 3ZIZ) exhibiting substituted amino-acids. A. Surface view of the catalytic cleft of *Pa*Man5A with mannotriose modelled in the −2 and −3 subsites and mannobiose modelled in the +1 and +2 subsites. The structures of GH5 from *T. reesei* and *T. fusca* in complex with mannobiose and mannotriose, respectively, were superimposed on the top of the structure of *Pa*Man5A to map the substrate-binding subsites. The two catalytic glutamate residues, E177 and E283, are coloured in red. The substituted amino-acids are labelled and coloured in yellow. B. Structural based sequence alignment of the region around position 311 (according to *Pa*Man5A numbering) from *Podospora anserina* (*Pa*Man5A), *Aplysia kurodai* (*Ak*Man, PDB 3VUP), *Mytilus edulis* (*Me*Man5A, PDB 2C0H), *Cellvibrio mixtus* (*Cm*Man5A, PDB 1UUQ), *Trichoderma reesei* (*Tr*Man5A, PDB 1QNR), *Lycopersicon esculentum* (*Le*Man4A, PDB 1RH9) and *Thermomonospora fusca* (*Tf*Man5, PDB 2MAN). Secondary structure elements, α-helix α7 and β-strand β8, are indicated below the sequences as a cylinder and an arrow, respectively. Strictly conserved residues, G311 and W315 (according to *Pa*Man5A numbering), are shown with a yellow and a grey background, respectively. C. Surface view of *Pa*Man5A rotated of about 90° along the horizontal axis. The front clipping plane has been moved in order to visualize the location of G311 inside the molecule. The zoom shows a compact hydrophobic core in the vicinity of G311.

**Figure 4:**
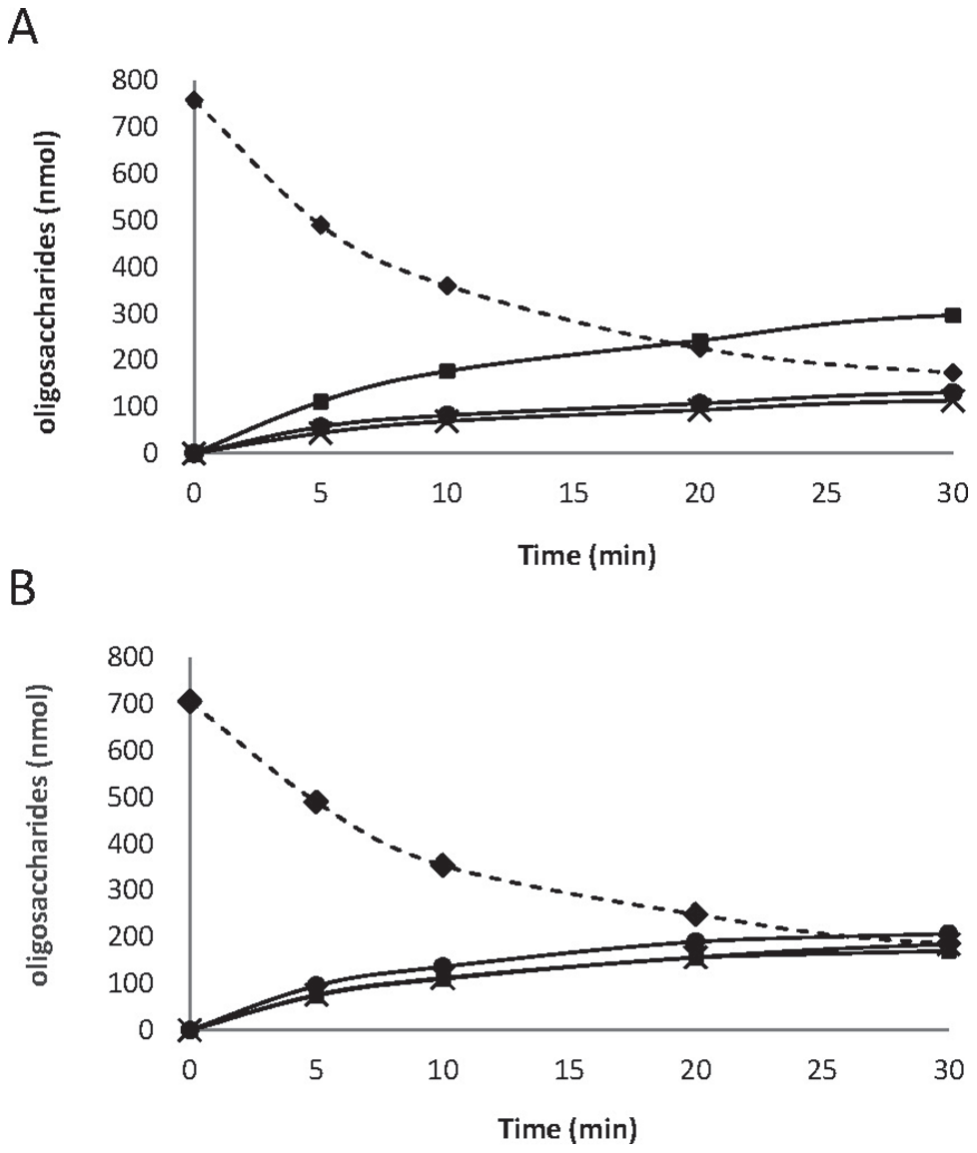
Progress curves of the manno-oligosaccharides generated by the wild-type *Pa*Man5A and the *Pa*Man5A-K139R/Y223H variant upon hydrolysis of mannohexaose. 18.2 nM of the wild-type *Pa*Man5A (A) and the *Pa*Man5A-K139R/Y223H variant (B) were incubated with 1 mM of mannohexaose in acetate buffer pH 5.2 at 40°C. The amount of each manno-oligosaccharide, i.e., mannobiose (full circles), mannotriose (full squares), mannotetraose (crosses), and mannohexaose (full diamonds), is indicated during the course of the reaction.

The localization of all of the mutated residues of the *Pa*Man5A mutants revealed that five mutated residues (W36R, K139R, Y223H, V256A/L, and Q316H) out of eight are located within the active site cleft and three mutations are located inside the enzyme core (I195T, G276V and G311S). Three mutations were identified in the *Pa*Man5A-W36R/I195T/V256A mutant, among which W36 and V256 that are located close to the catalytic site (Figure 3A) and I195T is located in the enzyme core (not shown). The W36 residue side chain lies at the bottom of the active site crevice and may contribute to the −4 subsite. Interestingly, the Val256 does not seem to be directly involved in the subsite organization of *Pa*Man5A, but mutation of this residue were found in two distinct variants (*Pa*Man5A-V256L/G276V/Q316H and *Pa*Man5A-W36R/I195T/V256A), indicating a possible hot spot for enzyme improvement to further explore. Concerning the *Pa*Man5A-K139R/Y223H mutant, analysis of the manno-oligosaccharides produced upon hydrolysis of M_5_ and M_6_ revealed a potential modification of substrate binding (Figure 4). Indeed, the wild type *Pa*Man5A is known to produce mainly M_3_ from M_6_, with small amounts of M_2_ and M_4_ [24]. In the case of *Pa*Man5A-K139R/Y223H, equimolar amount of M_2_, M_3_ and M_4_ were quantified (Figure 4), suggesting a displacement of the substrate binding from −3 to +3 subsites to −2 +4 subsites or −4 +2 subsites (Figure 3). Within the two mutations of mutant *Pa*Man5A-K139R/Y223H, it is interesting to note that the Y223 residue is located nearby the +3 subsite. The substitution of the aromatic ring, involved in stacking interaction with the sugar, may contribute to the modification of substrate binding at this position (Figure 3). The single mutation of *Pa*Man5A-G311S is located in the core of the enzyme within the start of β8-strand (Figure 3B). The G311 residue is strictly conserved among all of the GH5 mannanases of known structure. The G311 residue is conserved among GH5 mannanases within the start of the β8-strand and it should be noted that the last residue of this strand is the W315 residue (Figure 3B), which shapes the −2 subsite. The G311 residue is in close vicinity with a compact hydrophobic core made of residues F20, I60, I115, F170, F244, L280 and L313 (Figure 3C). It is difficult to determine the effect of this single substitution from a structural point of view, although we can hypothesize that the hydroxyl group of the serine residue may contribute to the modification of the β8-strand by interacting with the surrounding residues although circular dichroïsm analysis of the *Pa*Man5A-G311S mutant revealed a similar profile compared to the parental enzyme, suggesting that its folding was similar to the parental enzyme (data not shown). Therefore the single mutation *Pa*Man5A-G311S does not seem to modify the overall architecture of the enzyme but a slight movement of the β8-strand could move the W315 residue at the surface of the enzyme and explain the decreased K_M_.

**Figure 5:**
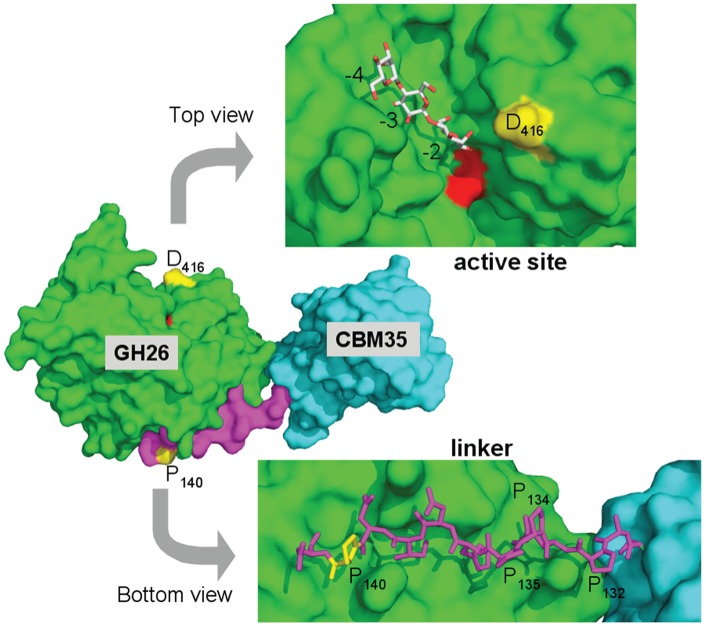
Structural view of *Pa*Man26A (PDB 3ZM8) exhibiting substituted amino-acids. The central panel shows a surface view of the entire *Pa*Man26A structure, which is composed of a carbohydrate binding module (CBM) belonging to the CBM35 family in cyan, a linker in violet and a catalytic domain belonging to the GH26 family in green. The two catalytic glutamate residues, E300 and E390, are coloured in red. The two substituted amino-acids, P140 and D416, are labelled and coloured in yellow. The top view represents the surface view of the catalytic cleft of *Pa*Man26A rotated about 90° along the horizontal axis with mannotriose modelled into the −2 to −4 subsites. The structure of GH26 from *C. fimi* in complex with mannotriose was superimposed on the top of the structure of *Pa*Man26A to map the substrate-binding subsites. The bottom view displays the *Pa*Man26A linker (from residue 131 to residue 141) in stick representation. The molecule has been rotated of about 90° along the horizontal axis and in the opposite direction compared to the top view. The proline residues of the linker are labelled.

One of the most remarkable findings of this study is the activity increase of the *Pa*Man26A-P140L/D416G mutant that contained only two mutations, i.e., one in the linker region (P140L) and one at the entrance of the active site (D416G) (Figure 5). The region from residue R133 to residue N141, which may be considered as the end of the linker, is tightly bound to the catalytic domain thanks to a dense network of hydrogen bonds and hydrophobic interactions [24]. Moreover, the *Pa*Man26A linker sequence contains four proline residues (P132, P134, P135 and P140) out of 12 residues that may confer rigidity to the modular enzyme [24]. In the *Pa*Man26A-P140L/D416G mutant, the P140 residue was substituted by a leucine residue, probably resulting in a decrease of linker rigidity that could partially explain the improved *k*_cat_/K_M_. The D416 amino-acid substitution is located at the edge of the catalytic cleft (Figure 5). The favourable mutation consisted in the removal of the carboxylic group since the D416 amino-acid was substituted by a glycine residue. We suggest that the lack of the carboxylic acid side chain may promote the entrance of the substrate into the active site.

## Conclusions

Activity improvement of fungal carbohydrate-active enzymes has typically met many obstacles, owing primarily to the lack of efficient expression systems and high-throughput screening methodologies. In this study, molecular engineering of two fungal mannanases was undertaken using random mutagenesis in the yeast *Y. lipolytica*. All of the selected mutants highlighted improved characteristics when compared to wild-type enzymes, thus validating the approach of mutagenesis and screening that were employed. Examination of the three-dimensional structures of *Pa*Man5A and *Pa*Man26A revealed several hot spots (i) around the active site of both mannanases, (ii) in the core region of *Pa*Man5A and (iii) in the linker region of *Pa*Man26A. The directed evolution strategy implemented in this study was very successful since a straightforward round of random mutagenesis yielded significantly improved variants that would have been difficult to predict using rational site-directed mutagenesis and structure-guided design.

## Methods

### Plasmids, strains and culture conditions

The growth media and culture conditions for the *Y. lipolytica* JMY1212 strain (Ura-, Leu+, ΔAEP, Suc+) have been previously described [30]. Briefly, the *Y. lipolytica* JMY1212 strain was cultured in YPD (10 g/l yeast extract, 20 g/l peptone, 20 g/l glucose) at 28°C, 130 rpm in baffled flasks or in Petri dishes containing YPD supplemented with agar (15 g/l). The plasmid used for expression in *Y. lipolytica* was JMP61 that displayed the *POX2* promoter for induction by oleic acid [31] and the Zeta recombination platform for controlled monocopy integration in *Y. lipolytica* genome. Subcloning of parental genes was performed using the TOPO TA kit (Invitrogen, Cergy-Pontoise, France) and TOP10 *E. coli* competent cells (Invitrogen).

### Construction of JMP61-*paman5a* and JMP61-*paman26a* plasmids and yeast transformation

*Pa*Man5A and *Pa*Man26A genes (*paman5a* [HM357135] and *paman26a* [HM357136], respectively) were amplified with primers listed in Table 3, starting from the pPICZαA-*paman5a* and the pPICZαC-*paman26a* plasmids [10]. Insertion of both genes in the JMP61 expression vector was performed using *Bam*HI and *Avr*II restriction sites. *Y. lipolytica* Zeta competent cells were prepared using the lithium acetate method as described in le Dall *et al* [32].

**Table 3:** List of primers used in the study.

Gene	PCR step	Primer name	Sequence (5′->3′)
*paman5a*	integration in JMP61	GH5JMP61F 5′	TTTGGATCCCTCCCCCAAGCACAA
		GH5JMP61R 3′	TTTCCTAGGCTACGCCGGGAGAGCATT
	mutagenesis PCR (PCR1)	JMP61EvDF	GCAGAAGCGATTCGGATCC
		PCR2r	GGAGTTCTTCGCCCACCC
	zeta platform PCR (PCR2)	PCR1d	GATCCCCACCGGAATTGC
		GH5EvDRev	GGCTGCTCCTCCACC
	integration in pPICZαA	GH5-InFuFOR	GGCTGAAGCTGAATTCCTCCCCCAAGCACAAGGT
		GH5-InFuR	GAGTTTTTGTTCTAGACCCCGCCGGGAGAGCATT
*paman26a*	integration in JMP61	JMP61-CBMGH26-F	TTTGGATCCAAGCCTTGTAAGCC
		GH26-JMP61-Rev	TTTCCTAGGCTAACTCCTCCACCCCTGAAT
	mutagenesis PCR (PCR1)	PCR1dtmutCBM-F	TTTCCAACCTCAACAACCCCAAC
		PCR2r	GGAGTTCTTCGCCCACCC
	zeta platform PCR (PCR2)	PCR1d	GATCCCCACCGGAATTGC
		GH26EvDRev	GGAGTAGAGCTTCTT
	integration in pPICZαA	GH26CBM-InFuFOR	GGCTGAAGCTGAATTCAAGCCTTGTAAGCCTCGT
		GH26InFuR	GAGTTTTTGTTCTAGACCACTCCTCCACCCCTGAAT
common	overlap PCR (PCR 3)	PCR1dL	CCGCTGTCGGGAACCGCGTTCAGGTGGAACAGG
		PCR2rL	CCGCACTGAGGGCTTTGTGAGGAGGTAACGCCG

Competent cells were immediately transformed with 500 ng *Not*I-linearized recombinant plasmid combined with 25 µg of salmon sperm DNA by heat shock at 39°C for 10 minutes and immediately recovered in 1.2 ml of 100 mM lithium acetate. Transformants were plated on YNB agar (1.7 g/l YNB, 10 g/l glucose, 5 g/l ammonium chloride, 2 g/l casamino acids, in 50 mM sodium–potassium phosphate buffer, pH 6.8, 17 g/l agar) and incubated at 28°C for 48 hours.

### Construction of mutagenesis libraries by error-prone PCR

Three PCR were carried out for each gene as shown in Figure 1 with primers listed in Table 3. Mutagenic PCR (PCR1) were performed using Genemorph II Random Mutagenesis Kit (Stratagene, La Jolla, CA) following the manufacturer's instructions using primers JMP61EvDF and PCR2r for *paman5a* and PCR1dtmutCBM-F and PCR2r for *paman26a*. The PCR contained 500 ng of parental gene, 200 µM dNTP, 0.25 µM primers and 2.5 units of Mutazyme. The PCR were made up to 50 µl and incubated at 95°C for 5 min and then at 95°C for 30 sec, 50°C for 30 sec, 72°C for 1 min and 30 sec for 40 cycles followed by 5 min at 72°C. PCR2 was carried out using HiFi polymerase (Invitrogen) to amplify the zeta platform without introducing any mutations. Primers used were PCR1d and GH5EvDRev for *paman5a* and PCR1d and GH26EvDRev for *paman26a*. PCR products were gel-extracted using Gel-Extraction kit (Qiagen, Courtaboeuf, France) and a third overlap PCR (PCR3) was subsequently carried out using gel-extracted products of PCR1 and PCR2 to rebuild the whole zeta fragment containing mutant genes. The primers used for PCR3 were PCR1dL and PCR2rL for the two constructions. The overlap PCR was carried out using i-Star Max II DNA polymerase (Intron Biotechnology, Boca Raton, FL).

PCR3 products were subsequently transformed into *Y. lipolytica* as described above and plated onto YNB in QTrays plates (Corning Corp, NY, USA) at a density of about 500 colonies par plate and incubated for 48 hours at 28°C.

### Screening of mutagenesis libraries

#### Agar plate-based screening

The Ura-positive *Y. lipolytica* transformants obtained from mutagenesis libraries were subsequently gridded on YNB agar medium containing oleic acid (1% v/v, Sigma) and Azo-galactomannan (0.2% w/v, Megazyme) using a QPixII colony picker (Genetix, Molecular Devices, Sunnyvale, CA, USA). The plates were further incubated at 28°C for 48 hours. Mannanase activity was visualized as clear halos around colonies within a blue background.

#### Liquid culture-based screening

Mannanase-positive colonies were picked on OmniTrays (Nunc, Thermo Fischer Scientific, Courtaboeuf, France) containing YPD agar and incubated for 48 hours at 328°C. Further screening was performed as described in [26]. Briefly, 96-well preculture plates containing 200 µl YPD were inoculated by individual colonies. The precultures were incubated at 800 rpm and 28°C overnight in a Microtron incubator (Infors HT, Switzerland). For expression of recombinant genes, 20 µl of each preculture were transferred in 1 ml of YTO medium (10 g/l yeast extract, 20 g/l% w/v tryptone, 2% oleic acid, in 50 mM phosphate buffer, pH 6.8) in 96-deep-well plates. Cultures were further incubated at 28°C with shaking at 800 rpm. After 4 days induction, the supernatants containing mutant enzymes were recovered by centrifugation (10 min, 3500 rpm) and endo-mannanase activity towards galactomannan was determined from DNS assay as described before [23]. Briefly, 10 µl of culture supernatant were incubated with 190 µl of 1% (w/v) galactomannan in 50 mM sodium phosphate buffer (pH 5) at 40°C. 80 µl of reaction mixture was recovered and reaction was terminated by addition of same volume of dinitrosalicylic acid reagent at 1% (w/v) in 96 well PCR plates. Samples were heated at 95°C for 10 min and DO_540_ was measured relative to a mannose standard curve (1 to 20 mM).

### Wild-type and mutant enzymes large-scale production

For heterologous production of *Pa*Man5A and *Pa*Man26A mutant proteins, the selected genes were amplified from genomic DNA using GH5-InFuFOR and GH5-InFuR for *Pa*Man5A and GH26CBM-InFuFOR and GH26-InFuR for *Pa*Man26A, respectively, listed in Table 3 and transferred into the pPICZαA plasmid using InFusion kit (Clontech, Takara). Resulting plasmids were transformed into *P. pastoris* and protein productions and purification were carried out as described before [10].

### Biochemical and biophysical characterization

Protein concentration was determined by using the Bio-Rad protein assay kit with bovine serum albumin as standard (Bio-Rad, Marnes-la Coquette, France) and UV absorbance at 280 nm. SDS-PAGE was performed in 10% (w/v) polyacrylamide gel (Bio-Rad) using a Pharmacia LMW electrophoresis calibration kit (GE Healthcare, Buc, France). Native IEF was carried out at 4°C in the Bio-Rad gel system, using pI standards ranging from 4.45 to 8.2. IEF gel was coloured with IEF staining solution (0.04% Coomassie Blue R250, 0.05% Cocrein Scarlett, 10% acetic acid, 27% isopropanol).

### Characterization and kinetic properties of individual enzymes

Determination of kinetic parameters on galactomannan was performed using DNS activity assay. Unless otherwise indicated, assay mixtures contained substrate and suitably diluted enzyme in sodium acetate buffer 50 mM, pH 5. Briefly, 1 µg of enzyme was mixed with 190 µl of galactomannan (Megazyme International, Wicklow, Ireland) using a range of substrate concentration from 1 to 20 mg.ml^−1^ (eight concentrations) and incubated at 40°C for 5 minutes. Reactions were performed in triplicate independent experiments. The reaction was stopped by the addition of 300 µl of 1% DNS reagent and samples were heated at 95°C for 10 minutes. DO_540_ was measured relative to a mannose standard curve (0 to 20 mM). One unit of endo-mannanase activity was defined as the amount of protein that released 1 µmol of sugar monomers per min. The kinetic parameters were estimated using weighted nonlinear squares regression analysis with the Grafit software (Erithacus Software, Horley, UK).

### Analysis of initial sugar release by HPAEC-PAD and kinetic parameters measurement

Monosaccharide and oligosaccharides generated after hydrolysis of manno-oligosaccharides (M_5_ and M_6_, Megazyme) were analysed using HPAEC-PAD. 10 µl of suitably-diluted enzyme were incubated at 40°C for various time lengths with 190 µl of 1 mM substrate in 50 mM acetate buffer pH 5.2. At each time point, 10 µl of reaction mixture were recovered and the reaction was terminated by the addition of 90 µl of 180 mM NaOH. For HPAEC analysis, 10 µl were injected and elution was carried out as described in [10]. Calibration curves were plotted using β-1,4-manno-oligosaccharides as standards from which response factors were calculated (Chromeleon program, Dionex) and used to estimate the amount of products released in test incubations. All the assays were carried out at least in duplicates. The specificity constants were calculated using the Matsui equation [24], [33].

## Supporting Information

Figure S1 **Isoelectrofocusing analysis of** ***Pa*** **Man5A and** ***Pa*** **Man26A variants.** 1: pI marker (values are on the left); 2: *Pa*Man26A wild-type; 3: *Pa*Man26A-P140L/D416G; 4: *Pa*Man5A wild-type; 5: *Pa*Man5A-V256L/G276V/Q316H; 6: *Pa*Man5A-W36R/I195T/V256A; 7: *Pa*Man5A-K139R/Y223H; 8: *Pa*Man5A-G311S.
(EPS)
